# Inference of age‐dependent case‐fatality ratios for seasonal influenza virus subtypes A(H3N2) and A(H1N1)pdm09 and B lineages using data from the Netherlands

**DOI:** 10.1111/irv.13146

**Published:** 2023-06-19

**Authors:** Scott A. McDonald, Anne C. Teirlinck, Mariette Hooiveld, Liselotte van Asten, Adam Meijer, Marit de Lange, Arianne B. van Gageldonk‐Lafeber, Jacco Wallinga

**Affiliations:** ^1^ Centre for Infectious Disease Control National Institute for Public Health and the Environment (RIVM) Bilthoven The Netherlands; ^2^ Nivel Utrecht The Netherlands; ^3^ Department of Biomedical Data Sciences Leiden University Medical Center Leiden The Netherlands

**Keywords:** case fatality rate, influenza A virus, H1N1 subtype, influenza A virus, H3N2 subtype, influenza B virus, Netherlands, seasons

## Abstract

**Background:**

Despite the known relatively high disease burden of influenza, data are lacking regarding a critical epidemiological indicator, the case‐fatality ratio. Our objective was to infer age‐group and influenza (sub)type specific values by combining modelled estimates of symptomatic incidence and influenza‐attributable mortality.

**Methods:**

The setting was the Netherlands, 2011/2012 through 2019/2020 seasons. Sentinel surveillance data from general practitioners and laboratory testing were synthesised to supply age‐group specific estimates of incidence of symptomatic infection, and ecological additive modelling was used to estimate influenza‐attributable deaths. These were combined in an Bayesian inferential framework to estimate case‐fatality ratios for influenza A(H3N2), A(H1N1)pdm09 and influenza B, per 5‐year age‐group.

**Results:**

Case‐fatality estimates were highest for influenza A(H3N2) followed by influenza B and then A(H1N1)pdm09 and were highest for the 85+ years age‐group, at 4.76% (95% credible interval [CrI]: 4.52–5.01%) for A(H3N2), followed by influenza B at 4.08% (95% CrI: 3.77–4.39%) and A(H1N1)pdm09 at 2.51% (95% CrI: 2.09–2.94%). For 55–59 through 85+ years, the case‐fatality risk was estimated to double with every 3.7 years of age.

**Conclusions:**

These estimated case‐fatality ratios, per influenza sub(type) and per age‐group, constitute valuable information for public health decision‐making, for assessing the retrospective and prospective value of preventative interventions such as vaccination and for health economic evaluations.

## 1 INTRODUCTION

Seasonal influenza contributes a substantial portion of the global infectious disease mortality burden, especially among older adults.^1^, ^2^, ^3^, ^4^ In countries with a temperate climate, influenza is one of the top‐ranked infectious diseases in terms of health burden when measured as disability‐adjusted life years (DALY).^5^, ^6^ Despite the relatively high disease burden of influenza, data regarding the case‐fatality ratio (cfr) following symptomatic infection are scarce. In one of the few studies from which an influenza‐attributable cfr can be extracted, 0.19% of a study population of 141,000 influenza patients who consulted their general practitioner (GP) died within 30 days, compared with 0.06% of matched controls.^7^

During the recent 2009/2010 influenza A(H1N1)pdm09 pandemic, initiatives to compile surveillance and seroprevalence data of sufficient quality to estimate cfrs per age‐group were undertaken,^8^, ^9^, ^10^ but comparable figures for influenza A(H1N1)pdm09 in post‐pandemic years, A(H3N2) and the B lineages are lacking. The reason is that determining both numbers of cases and deaths attributable to influenza is extremely challenging without data sources specifically designed to compile these figures. Influenza A(H3N2) virus infection is suspected to lead to a more severe disease course (including mortality) compared with influenza A(H1N1) virus,^11^ with influenza B possibly falling in between,^12^ although there is actually little supporting evidence for a consistent difference in clinical severity between influenza A and B.^13^, ^14^, ^15^

In the Netherlands, estimates of incidence rates of symptomatic influenza infection are routinely computed using evidence synthesis methods applied to sentinel surveillance of influenza‐like illness (ILI), virological testing and other data sources,^16^, ^17^ and annual estimates of influenza‐attributable mortality are produced from registered cause of death data using established additive regression approaches.^18^ Our objective was to combine these two sets of estimates within a statistical modelling framework to infer the cfrs of seasonal influenza per age‐group and (sub)type. These case‐fatality estimates can be used to improve the accuracy of disease burden calculations.^5^

## 2 METHODS

We defined our analysis period to consist of the nine winter seasons from 2011/2012 through 2019/2020. Seasons are defined as week number 40 through week number 20 of the following year. This period fell between the 2009–2010 influenza A(H1N1)pdm09 pandemic and the COVID‐19 pandemic beginning in March 2020 in the Netherlands. During the selected period, the coverage of the various surveillance systems for respiratory infections can be considered to be reasonably stable. We conducted analyses for the following set of 19 age‐groups (all consisting of 5 years, except for the oldest and the two youngest groups): <1 year, 1–4 years, 5–9, 10–14, 15–19, …, 80–84, 85 years and older.

### 2.1 Estimation of the incidence of symptomatic infection per age‐group and influenza (sub)type

An evidence synthesis approach, combining GP‐based sentinel surveillance for ILI,^19^ laboratory testing of a weekly subset of GP‐consulting ILI patients for influenza virus, including subtyping of all detected influenza A viruses and lineage determination of all detected influenza type B viruses,^16^ and internet survey‐based data on health care seeking behaviour, is routinely deployed^16^, ^17^ to estimate the incidence rate of symptomatic influenza infection per age‐group (defined as <5, 5–14, 15–44, 45–64 and 65+ years; age categories defined based on virological testing data) and per winter season. We multiplied these incidence rates by the 5‐year age‐group population sizes^20^ to obtain season‐specific estimates of the number of symptomatic infections per narrow age‐group (making the simplifying assumption that ILI rates from sentinel surveillance are applicable to the 5‐year age‐groups within each broader age‐group) (Figure S4). Furthermore, the laboratory testing of the subset of ILI patients provides quantitative information on the season‐specific circulating subtypes/lineages, namely, the proportion of samples per influenza virus A subtype (H3N2, H1N1) and B lineage (Victoria, Yamagata). This allows the age‐ and season‐specific estimates of symptomatic incidence to be further broken down into three categories: influenza A(H3N2), influenza A(H1N1)pdm09 and influenza B (we pool both B lineages due to their relatively small individual contributions over our analysis period).

### 2.2 Influenza mortality attribution

We adopted an ecological modelling approach frequently used by previous research for attribution of influenza‐attributable deaths,^18^, ^21^, ^22^, ^23^ the fundamental assumption of which is that seasonal variability in mortality can be (partly) explained by temporal variation in the reporting incidence of viral or bacterial respiratory disease‐causing pathogens.

#### 2.2.1 Data sources

Attribution of influenza‐attributable deaths requires data on the weekly number of deaths from a respiratory cause. Statistics Netherlands registers all deaths in the Netherlands; publicly available weekly all‐cause mortality data, stratified into the ‘broad’ age‐groups <65, 65–79 and 80+ years were obtained for our analysis period.^24^ Additionally, for the years 2011–2013, we made use of a dataset analysed in our previous study,^25^ in which respiratory deaths (defined as deaths with an underlying respiratory cause of death [ICD‐10: J00‐J99]) were separately coded and were stratified by narrower age‐groups (<55 years, 55–59 … 85+ years). This shorter time series informed the estimation of respiratory deaths from weekly all‐cause deaths. Because of uncertainty regarding the contribution of SARS‐CoV‐2 infection versus influenza virus or other pathogens to respiratory mortality from approximately ISO week 12 of 2020 (COVID‐19 deaths began to rise in Week 11 of 2020^26^), we truncated all‐cause mortality data for the 2019/2020 season at Week 11 (for consistency, we truncated all data sources involved in the estimation of symptomatic infection incidence for the 2019/2020 season also at Week 11). To reconstruct the number of respiratory deaths per 5‐year age‐group within each broad age‐group for the remaining years (period from 2014 through week 11 of 2020), we derived both sets of distributions (e.g., the proportions 80–84 and 85+ years within the available 80+ years data; the proportion of all‐cause deaths with an underlying respiratory cause) from this earlier dataset. We calculated these distributions per ISO week number (thus aggregating over year, because the proportion of respiratory deaths among all‐cause mortality could exhibit seasonality, which could also influence the distribution of deaths occurring among the component sub‐groups of a broad age‐group), using the last 4 years of available data, that is, 2010–2013.

Since 1989, a centralised database (the Weekly Surveillance System of the Dutch Working Group on Clinical Virology) compiles routine weekly counts of positive laboratory test results for a range of respiratory pathogens from between 17 and 21 virological laboratories in the Netherlands; data are mainly from hospitalised patients.^27^ With the use of these data, we extracted weekly counts of laboratory diagnoses for the following viral and bacterial agents: influenza virus type A, influenza virus type B, respiratory syncytial virus, rhinovirus, parainfluenza virus and *Mycoplasma pneumoniae* (set consistent with that used in van Asten et al.^18^), for the seasons 2011/2012 through 2019/2020.

#### 2.2.2 Additive regression modelling to infer influenza‐attributable deaths

With the use of additive regression modelling, we estimated the weekly number of deaths attributable to influenza after adjusting for the co‐circulation of other pathogens and other factors via linear regression techniques. We fitted separate Poisson regression models with an identity link function to allow an additive interpretation of model coefficients, to the weekly respiratory mortality data for each age‐group (<55 through 85+ years). As laboratory virological surveillance data were not available stratified by age, each age‐group specific additive regression model adjusted for the total reported positive samples per pathogen (i.e., all laboratory weekly surveillance data were used to develop each the regression model for each age‐group). This modelling method is more fully described in previous publications.^18^, ^25^

#### 2.2.3 Covariates and model selection

Besides the above‐named candidate respiratory pathogens (influenza A virus—coded using separate variables for each season, to capture seasonal variation in severity, influenza B virus, respiratory syncytial virus, rhinovirus, *M. pneumoniae* and parainfluenza virus), we explored extreme temperature, defined using daily temperature data recorded at the De Bilt (centrally located in the Netherlands) weather station and available online,^28^ and then coding low extreme temperature using the function max(0, 5–*T*), and high extreme as max(0, *T*–17), where *T* is the mean weekly temperature, in degrees Celsius.^18^, ^29^ Temperatures as thus treated as ‘extreme’ if below 5°C or above 17°C.

Next, we conducted a structured model selection procedure separately for each age‐group, which involved first entering linear and quadratic trend terms, then testing the impact of 0‐ to 4‐week lags between weekly surveillance reports of influenza A and B viruses and the other co‐circulating pathogens and mortality, selecting the lagged pathogen term that led to the largest AIC reduction, then adding terms for low and high extreme temperature terms, and finally adding trigonometric terms to account for the assumed sinusoidal‐shaped background mortality (for further details see van Asten et al. and McDonald et al.^18^, ^25^).

To estimate the number of influenza‐attributable deaths among 5‐year age‐groups below 55–59 years, we used a different approach that was not sensitive to the low counts in these age‐groups. For this, we adopted the method implemented in DALY computation software^30^ developed for the Burden of Communicable Diseases in Europe (BCoDE) project,^5^ which ‘redistributes’ the total influenza‐attributable deaths according to a country‐aggregated data source, namely, the observed age‐distribution of influenza‐coded deaths based on ICD‐10 codes, averaged over four countries (Estonia—Estonian Insurance Database, Germany—Federal Statistical Office, Italy—Italian National Bureau of Statistics and Netherlands—Statistics Netherlands) and over the 4‐year period 2005–2008 (2006–2007 only for Italy). We applied the age‐group specific proportions from this aggregate data source (among <55 years only) to the total number of estimated deaths in the <55 years age‐group for each season, to estimate the number of influenza‐attributable deaths per 5‐year age‐group per season. This aggregate data source indicates a decreasing proportion with decreasing age‐group until 5–9 years (see Figure S3); the proportion for 1–4 years is higher than for 5–9 years and higher still for <1 year; this pattern is consistent with clinical data on rates of in‐hospital mortality among children hospitalised with confirmed influenza virus infection.^31^

### 2.3 Inference of cfrs for influenza A(H3N2), A(H1N1)pdm09 and influenza B

(Sub)type‐aggregated cfrs are calculated by simply dividing the total estimated number of influenza‐attributable deaths per age‐group by the total estimated number of symptomatic infection cases (i.e., persons with ILI) per age‐group. Given that our data (estimated influenza‐attributable deaths, derived using additive regression) are not stratified by influenza (sub)type, the task is to find (sub)type‐dependent cfrs that when multiplied by the (sub)type‐specific symptomatic incidence and summed over (sub)type provide a good fit to the observed total number of influenza deaths per season. This is an inferential task, for which a solution can be found using Markov chain Monte‐Carlo (MCMC) sampling methods, provided there is sufficient variation in the distribution of circulating type and subtype (within influenza A) over seasons. For example, the presence of multiple seasons with a relatively high proportion of circulating influenza A(H3N2) and multiple seasons with a relatively high proportion of influenza B lineages will help identify these two cfrs.

Because of the relatively low influenza‐attributable mortality for age‐groups younger than 55 years (see Table 1), we used the inference approach to estimate cfrs for the seven age‐groups from 55–59 through 85+ years and a simple extrapolation approach for the younger age‐groups (see below). We implemented the below set of equations in JAGS^32^ and took 8000 samples from the posterior distributions for age‐specific cfrs after discarding 15,000 iterations as ‘burn‐in’. Note that as computation is carried out within a Bayesian framework, taking the 2.5% and 97.5% quantiles of the posterior distribution yields 95% credible intervals (CrIs). While the 95% intervals for (sub)type‐aggregated cfrs were estimated by propagating the uncertainty in influenza‐attributable mortality and symptomatic incidence in JAGS, uncertainty in the parameters incidence, mortality and (sub)type‐positive proportions could not be supplied as prior distributions in the inferential model. This is because the ‘best‐fitting solution’ (i.e., converged‐upon posterior distribution) may also adjust, sometimes drastically, of the posterior distributions for three parameters, which results in the inference of unrealistic cfrs.

**TABLE 1:** Estimated incident cases of symptomatic influenza (shown to four significant digits), reconstructed deaths with a respiratory condition as the underlying cause of death and influenza‐attributable respiratory deaths in the Netherlands, aggregating over nine seasons 2011/2012 through 2019/2020, with resulting case‐fatality ratios. CI = confidence interval.

Age‐group	Symptomatic influenza incidence (95% CI)	Total reconstructed respiratory deaths	Influenza‐attributable deaths (95% CI)	Case‐fatality ratio (95% CI)
(All ages)	4,676,000 (4,551,000–4,804,000)	121,729	5585 (5563–5608)	0.120% (0.116–0.123%)
85+ years	70,680 (62,780–79,680)	53,042	2999 (2982–3016)	4.28% (3.77–4.78%)
80–84	79,540 (70,310–89,430)	26,034	1105 (1095–1115)	1.40% (1.24–1.57%)
75–79	111,900 (99,490–125,600)	16,760	546 (540–552)	0.49% (0.44–0.50%)
70–74	152,300 (134,600–171,600)	10,545	418 (412–423)	0.27% (0.24–0.31%)
65–69	203,000 (179,300–228,900)	6770	220 (216–224)	0.11% (0.10–0.123%)
60–64	256,400 (237,500–275,700)	3847	100 (97–103)	0.039% (0.036–0.042%)
55–59	282,100 (261,600–303,800)	2261	81 (78–83)	0.029% (0.026–0.031%)
50–54	306,500 (284,100–330,500)	484	23 (22–24)	0.007% (0.007–0.008%)
45–49	310,200 (287,300–334,300)	494	23 (23–24)	0.008% (0.007–0.008%)
40–44	277,200 (255,100–301,200)	364	17 (17–18)	0.006% (0.006–0.007%)
35–39	249,700 (229,100–271,200)	237	11 (11–12)	0.005% (0.004–0.005%)
30–34	251,300 (230,900–273,600)	106	5 (5–5)	0.002% (0.002–0.002%)
25–29	262,700 (241,100–285,600)	98	5 (4–5)	0.002% (0.002–0.002%)
20–24	261,200 (240,400–284,300)	106	5 (5–5)	0.002% (0.002–0.002%)
15–19	250,000 (229,700–272,100)	76	4 (4–4)	0.001% (0.001–0.002%)
10–14	417,500 (368,900–469,400)	86	4 (4–4)	0.001% (0.001–0.001%)
5–9	392,300 (346,400–443,200)	76	4 (4–4)	0.001% (0.001–0.001%)
1–4	431,200 (365,100–507,400)	161	8 (7–8)	0.002% (0.001–0.002%)
<1	104,300 (88,500–122,200)	182	9 (8–9)	0.008% (0.007–0.010%)

We model the observed data, *Z*_*a,i*_, as the summation of the (unknown) (sub)type‐specific influenza deaths (i.e., *Y*_H3N2,a,i_ + *Y*_H1N1,a,i_ + *Y*_B,a,i_) using the *sum* sampler in JAGS (Figure S5 provides a directed acyclic graph of the statistical model). We use the index *i* to refer to the season of the analysis period, the index *a* to refer to the 5‐year age‐group and the index *s* to refer to the (sub)type. *Y*_*s,a,i*_ is therefore the number of respiratory deaths in season *i* and age‐group *a* among persons infected with influenza (sub)type *s*.

(1) $$Z_{a,i}=\sum_{s}Y_{s,a,i}.$$

The unknown number of (sub)type‐specific influenza deaths is assumed to follow a Poisson distribution:

(2) $$Y_{s,a,i}\sim \text{Poisson}\left(\lambda_{s,a,i}\right).$$

The Poisson rate parameter, $\lambda_{s,a,i}$, is a function of the (sub)type‐ and age‐dependent cfr (*cfr*) and the constants: the proportion of each (sub)type circulating per season (*π*_*s,i*_) and the estimated symptomatic incidence over the season (*SI*):

(3) $$\lambda_{s,a,i}={\mathrm{cfr}}_{s,a}*\pi_{s,i}*{\mathrm{SI}}_{a,i}.$$

The cfr is defined in turn to be a multiplicative function of a (sub)type‐specific cfr and age; we therefore define log (*cfr*) to be an additive function of a (sub)type‐specific constant term ( $\beta_{s}$) and the age‐effect coefficient (*μ*) multiplied by the reference age (in years; we define the reference age [age.ref] as the lower bound of the age‐group minus 50). This enforces an identical exponential relationship between cfr and (absolute) age for each (sub)type; this relationship is illustrated in a plot of the type‐aggregated data (Figure S1, ages 55–59 and older):

(4) $${\mathrm{cfr}}_{s,a}=e^{\beta_{s}+\mu *\mathrm{age}.{\mathrm{ref}}_{a}}$$

Given the Bayesian framework, prior distributions are required for stochastic parameters. Therefore, we assign vague normal priors to $\beta_{s}$ and μ, where the precision (1/*sd* ^2^) = 0.001

$$\beta_{s}\sim \text{Normal}\left(0,{\mathrm{sd}}^{2}\right).$$

$$\mu \sim \text{Normal}\left(0,{\mathrm{sd}}^{2}\right).$$

Finally, extrapolation of (sub)type‐specific cfrs downwards to the age‐groups 50–54 years and younger was conducted by multiplying the cfr estimated for 55–59 years by the separate age‐effects for 50–54 through 10–14 years and for 5–9 years through <1 year, which were separately estimated via log‐linear regression analysis of the type‐aggregated data (Figure S1).

Additive regression modelling was conducted in the R statistical programming environment, version 4.1.3^33^ and inference of cfrs using MCMC sampling within JAGS,^32^ accessed via the runjags package.^34^ JAGS code for estimation of cfrs is provided in the Supporting Information (Code S1 for [sub]type‐specific and Code S2 for aggregated over [sub]type).

## 3 RESULTS

**FIGURE 1:**
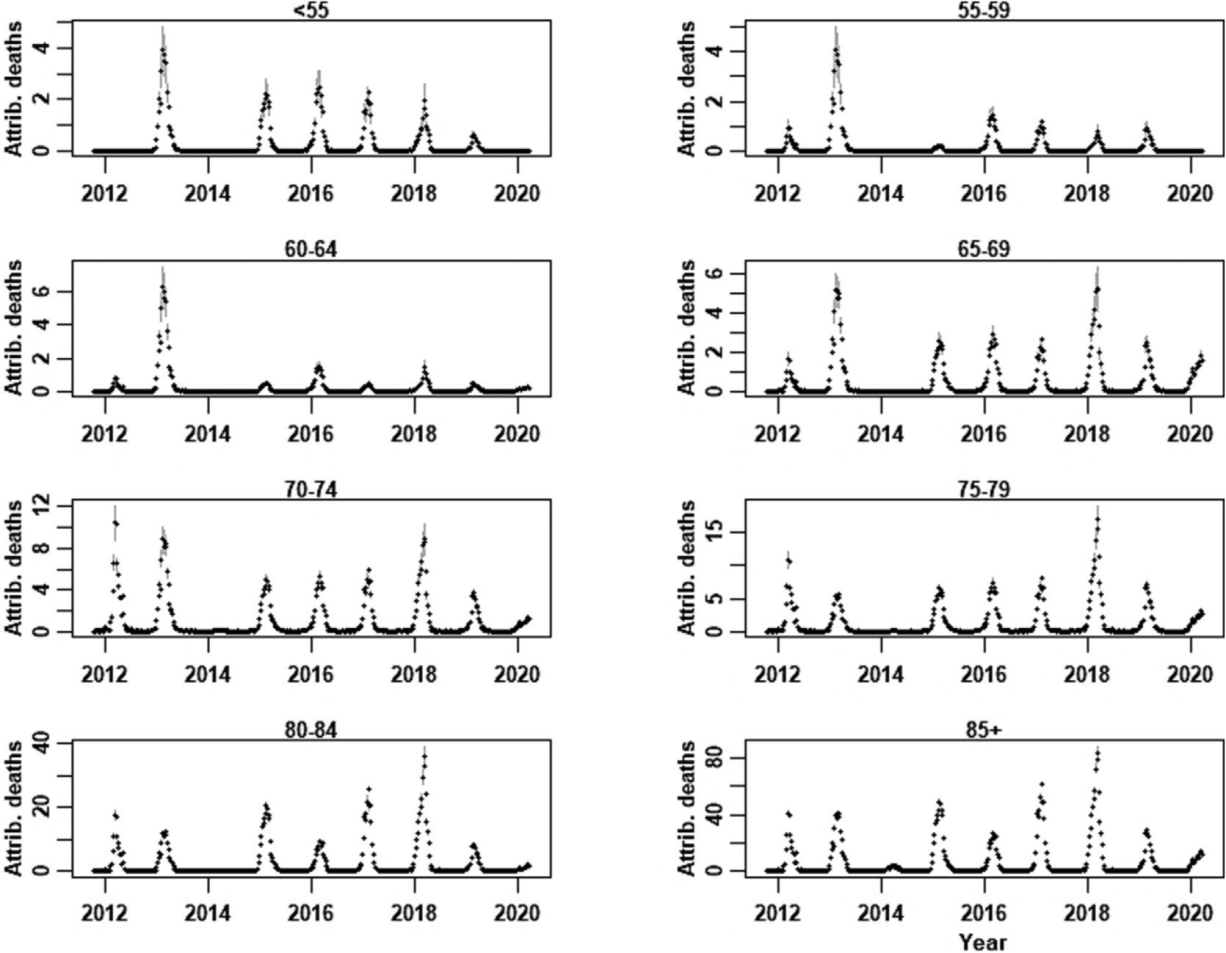
Additive regression‐modelled influenza‐attributable deaths in the Netherlands per age‐group (<55 years, 55–59 through 85+ years), for the seasons 2011/2012 through 2019/2020; 95% confidence intervals are indicated in grey.

A total of 1,241,193 all‐cause deaths were registered by Statistics Netherlands over our 9‐season analysis period. We reconstructed 121,729 (9.8% of the total) deaths due to an underlying respiratory cause; total respiratory deaths ranged from 2470 for <55 years to 53,042 for 85+ years (Table 1). The total number of additive‐regression‐modelled influenza‐attributable deaths ranged from 52 (in the 2013/2014 season) to 1260 in the 2017/2018 season (when influenza B/Yamagata was dominant) and varied widely by age‐group, with the highest and lowest estimates obtained for the 85+ years (*n* = 2999; 95% CI: 2982–3016) and 55–59 years (*n* = 81; 95% CI: 78–83) age‐groups, respectively (Table 1), which was consistently observed across seasons (Figure 1).

The re‐distribution of the total respiratory deaths and total modelled influenza‐attributable deaths for the <55 years among the 12 younger age‐groups is provided in Table 1. Over all seasons, an estimated 117 (95% CI: 115–118) influenza‐attributable deaths were estimated for persons younger than 55 years; for the age‐groups <1 year through 30–34 years, this estimate was extremely low, at between 4 and 9 deaths over the nine seasons. Table 1 also shows the estimated cfrs per age‐group, aggregating over (sub)type and season; this ranged from 4.28% (95% CI: 3.77–4.78%) for 85+ years to a low of 0.001% (95% CI: 0.001–0.001%) for persons aged 5–14 years.

**FIGURE 2:**
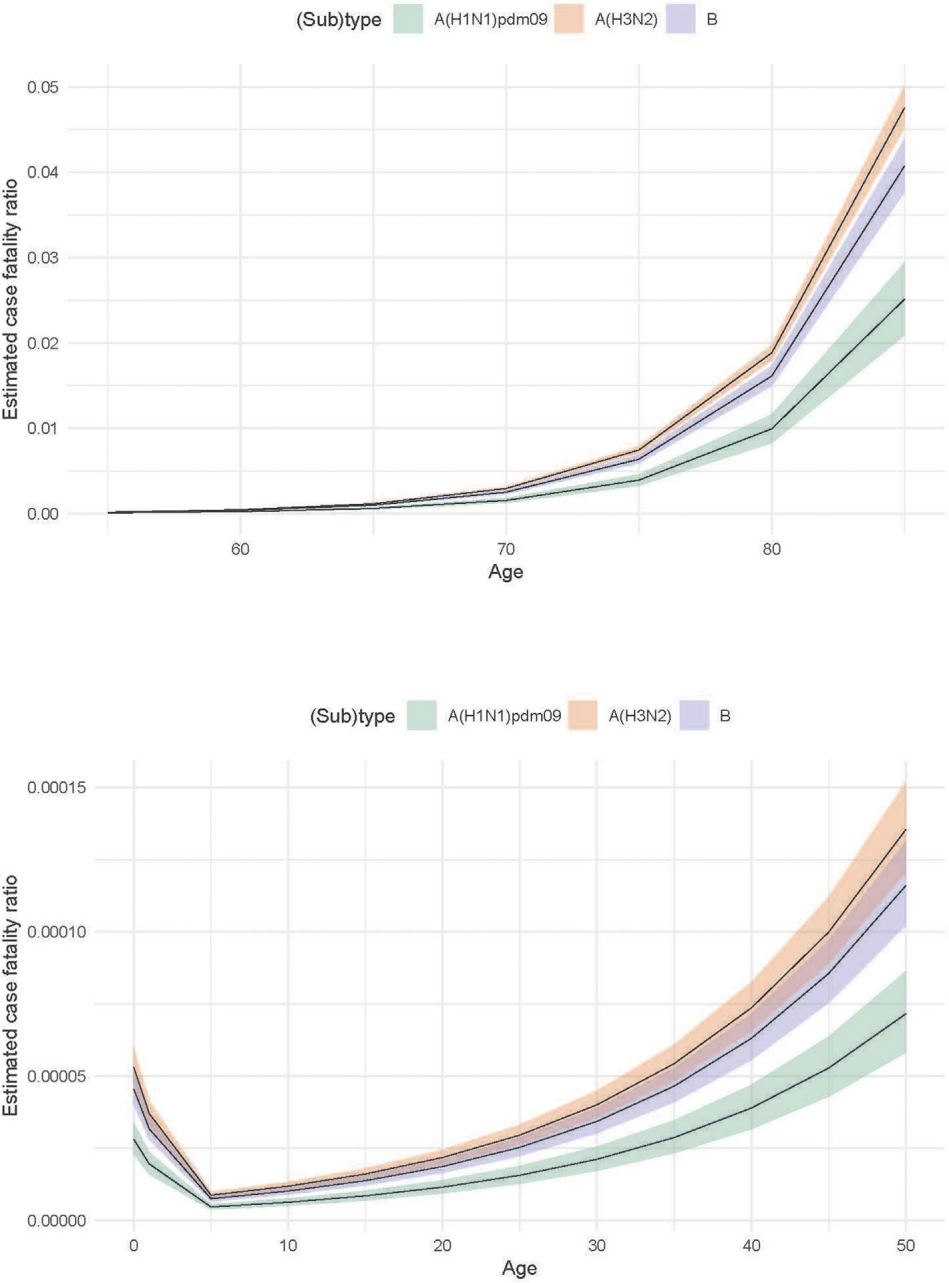
Inferred influenza case‐fatality ratios in the Netherlands per 5‐year age‐group, based on data from seasons 2011/2012 through 2019/2020: (upper panel) age‐groups 55–59 through 85+ years, (lower panel) age‐groups <1 year through 50–54 years. Note difference in *y*‐axis scales.

The Bayesian inference method applied to the age‐groups 55–59 through 85+ years yielded the highest age‐aggregated cfrs for influenza A(H3N2) (0.582%; 95% CrI: 0.563–0.601 in Section 4), followed by influenza B (0.486%; 95% CrI: 0.457–0.514) and influenza A(H1N1)pdm09 (0.283%; 95% CrI: 0.240–0.326). The highest cfr was estimated for 85+ years for all (sub)types (posterior median estimates of 4.76%, 4.08% and 2.51%, for A(H3N2), B and A(H1N1)pdm09, respectively) (Table 2 and Figure 2). The cfr was 2–3 orders of magnitude times smaller for the 55–59 years age‐group (0.018%, 0.016% and 0.010%, for A(H3N2), B and A(H1N1)pdm09, respectively). Extrapolated cfrs for the age‐groups 50–54 years and below are shown in Figure 2.

**TABLE 2:** Age‐group specific case‐fatality estimates for age‐groups 55–59 through 85+ years, separately estimated for influenza A(H3N2), influenza A(H1N1)pdm09 and influenza B, based on Netherlands data from the nine seasons 2011/2012 through 2019/2020. Total (aggregating over [sub]type) case‐fatality ratios from Table 1 are also shown. CI = confidence interval.

Age‐group		Estimated (sub)type‐specific case‐fatality ratio (%) (median, 95% CrI)		
	Total (95% CI)	A(H3N2)	A(H1N1)pdm09	Influenza B
85+ years	4.28% (3.77–4.78%)	4.76 (4.53–5.01)	2.51 (2.09–2.94)	4.08 (3.77–4.39)
80–84	1.40% (1.24–1.57%)	1.89 (1.80 1.98)	1.00 (0.83–1.16)	1.61 (1.49–1.73)
75–79	0.49% (0.44–0.55%)	0.75 (0.71–0.79)	0.39 (0.33–0.46)	0.64 (0.59–0.69)
70–74	0.27% (0.24–0.31%)	0.30 (0.28–0.32)	0.16 (0.13–0.18)	0.25 (0.23–0.28)
65–69	0.11% (0.10–0.12%)	0.12 (0.11–0.13)	0.062 (0.051–0.073)	0.10 (0.09–0.11)
60–64	0.039% (0.036–0.042%)	0.046 (0.042–0.051)	0.025 (0.020–0.029)	0.040 (0.036–0.045)
55–59	0.029% (0.026–0.031%)	0.018 (0.016–0.021)	0.010 (0.008–0.012)	0.016 (0.014–0.018)

**FIGURE 3:**
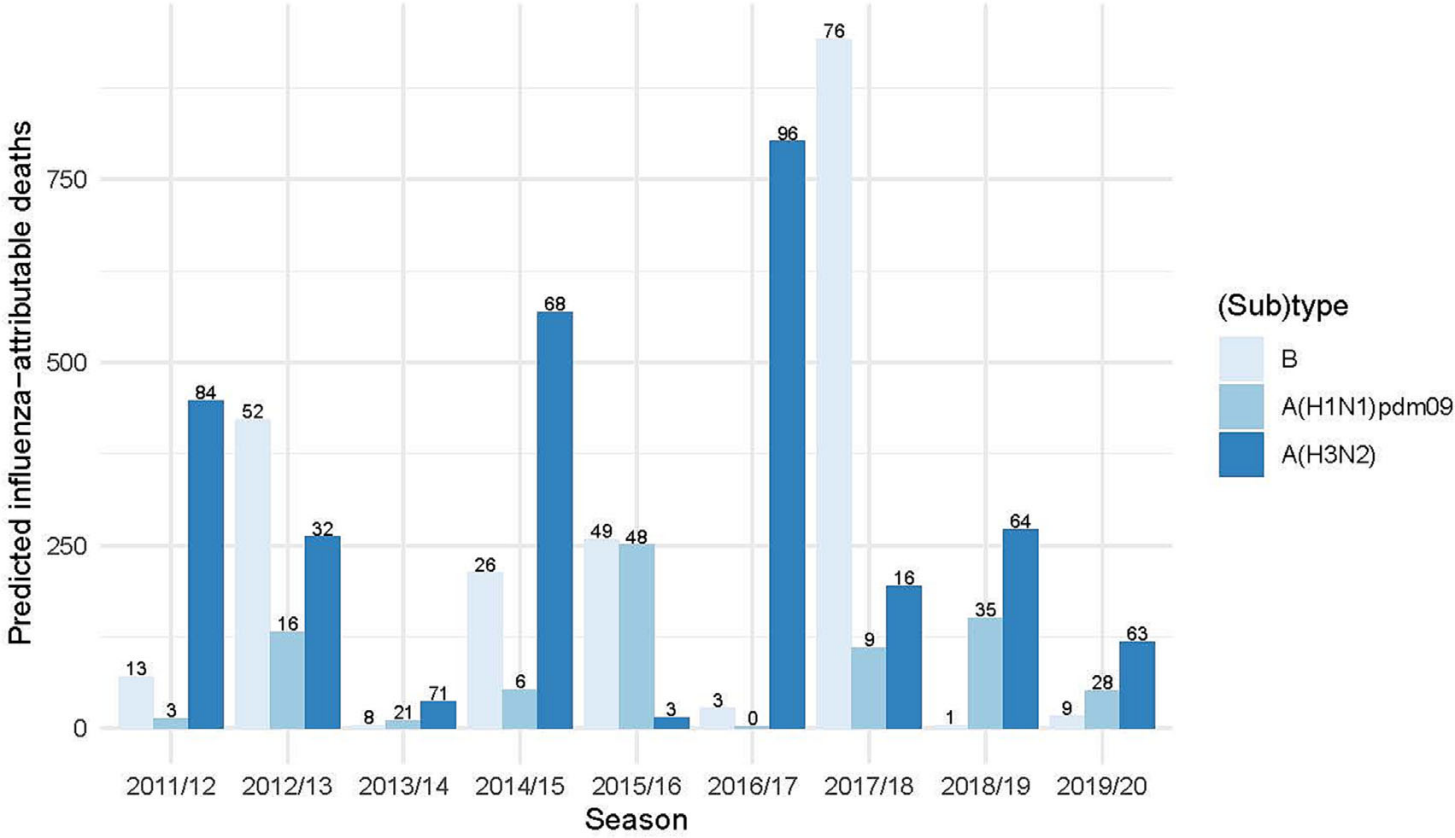
Fitted influenza‐attributable deaths, per season and (sub)type (aggregated over age‐groups 55–59 years and older). Numbers over each bar indicate the predicted percentage of deaths per (sub)type within each season.

The predicted number of influenza deaths per (sub)type and season (i.e., the quantity estimated using Equation 2) is shown in Figure 3. Seasons with relatively high influenza A(H3N2) circulation, such as 2011/2012 and 2016/2017 (see Figure S4), also have relatively high mortality attributable to this subtype; in contrast, seasons with relatively high influenza A(H1N1)pdm09 circulation (e.g., 2015/2016 and 2018/2019, at 60% and 51%, respectively) have relatively low estimated influenza A(H1N1)pdm09 mortality (and low mortality in general).

Model validity can be ascertained by comparing the total predicted influenza deaths (computed using the inferred cfrs) with the regression‐modelled influenza‐attributable deaths, per season and age‐group (Figure S2). Predicted mortality was a reasonable match to attributable mortality in all seasons, except for two seasons in which attributable deaths were under‐predicted. These were 2011/2012, visible for the 85+ years age‐group, and 2012/2013, for the 55–59 through 70–74 and 85+ years age‐groups.

For the age‐range 55–59 through 85+ years, the age‐effect was estimated at 1.204 (95% CrI: 1.199–1.208) (or 20% per year of age). Therefore, the number of years of age for the case‐fatality risk to double is calculated as log(2)/log (age‐effect) or 3.7 years.

## 4 DISCUSSION

By combining routinely calculated season‐ and age‐specific incidence rates of symptomatic influenza infection with estimates of influenza‐attributable respiratory deaths, we inferred age‐group dependent cfrs for influenza A(H3N2), influenza A(H1N1)pdm09 and influenza B lineages over the period 2011/2012 through 2019/2020 in the Netherlands. cfr estimates for the 85+ years age‐group were the highest, at 4.76% (95% CrI: 4.52–5.01%) for influenza A(H3N2), followed by influenza B at 4.08% (95% CrI: 3.77–4.39%) and influenza A(H1N1)pdm09 at 2.51% (95% CrI: 2.09–2.94%).

Unlike for COVID‐19, for which much scientific effort has been aimed at determining the infection‐fatality ratio, or the risk of death following infection,^35^, ^36^ cfrs for seasonal influenza have received far less attention—mainly due to unavailability of adequate data. In a UK study involving 141,293 persons who had an influenza‐associated GP consultation in the period 1991–1996, 0.19% of cases died within 30 days compared with 0.06% of matched controls,^7^ suggesting an overall influenza‐attributable cfr of 0.13%. Even though this study was based on data from 25–30 years ago and was restricted to persons who consulted their GP, the cfr estimated using our ecological design (aggregating over [sub]type and age‐group) based on data from nine recent seasons was comparable, at 0.12%.

During the influenza A(H1N1)pdm09 pandemic, for which adequate surveillance data exist, the all‐ages cfr from UK datasets was estimated by Donaldson et al.^8^ at 0.026% (95% CI: 0.011–0.066%); per age‐group estimates ranged from a low of 0.011% (0.003–0.036%) for 5–14 years to 0.98% (0.30–3.2%) for 65+ years. Based on US data from April through June 2009, Presanis et al.^9^ estimated the all‐ages cfr for influenza A(H1N1)pdm09 to be slightly higher, at 0.048% (95% CrI: 0.026–0.096%); per age‐group estimated ranged from 0.010% (0.003–0.031%) for 5–17 years to 0.159% (0.066–0.333%) for the 18–64 years age‐group. Our influenza A(H1N1)pdm09 cfr estimate for post‐pandemic seasons for persons aged 65+ years (0.54%, 95% CrI: 0.46–0.62%) is considerably lower than reported by Donaldson et al.; possible explanations include a diminishing of virulence or a build‐up of natural immunity subsequent to 2009–2010 and the availability of effective vaccines against a fatal outcome.

Among persons aged 55 through 85 years, we estimated an increased influenza‐attributable case‐fatality of 20% per year of age, which corresponds to a doubling of risk approximately every 4 years of age. For comparison, the population‐level mortality rate due to COVID‐19 during the first 16 weeks of the 2020 epidemic in England and Wales doubled with every 6 years of age.^37^

There were the small numbers of B/Victoria and B/Yagamata lineages detected through virological testing in most seasons of our analysis period; therefore, we aggregated the two influenza B lineages together, as the method cannot infer a cfr for a relatively rare subtype/lineage. Although the severe 2017/2018 influenza B/Yagamata‐dominant season led to considerable influenza‐attributable mortality, the inclusion of this season does not bias the cfr for influenza B, because the method takes advantage of variation in the (sub)type distribution across seasons and is not sensitive to absolute proportions. Nevertheless, because the inferred cfrs for influenza B are based on the aggregated data, a potential difference in severity between the two lineages cannot be recovered. As B/Yagamata has not been detected since April 2020 and appears to be extinct,^38^ the generalisability of our influenza B case‐fatality estimates to future seasons with appreciable circulation of B/Victoria is unknown.

We highlight the following limitations of our analysis. The inferred cfrs are sensitive to season‐specific vaccine effectiveness (VE)—seasonal variation in VE due to strain variability and vaccine (mis)match^26^—which, although the model provided real‐world case‐fatality estimates for our analysis period, these may not generalise to future situations with improved vaccines. As such, some portion of the between‐(sub)type difference we observed may be attributable to these factors. The age effect on cfrs was constrained to be identical for the three (sub)types, when in reality, ratios could reflect an interaction between (sub)type and age‐group. This is because mortality risk is influenced by both vaccine match and VE (for age‐groups with high vaccine uptake) and by pre‐existing immunity, for instance if a particular (sub)type circulated widely within the same age‐group or within age‐groups that have high contact rates with this age‐group, in the prior season(s). Related to this point, we estimated cfrs for age‐groups below 55–59 years using an extrapolation approach based on piecewise age‐effects and (sub)type‐specific cfrs derived from analysis of the 55–59 through 85+ years age‐groups. This was necessitated by the low number of influenza‐attributable deaths per season estimated for younger persons, which could not be fitted in the current framework.

Although influenza‐attributable deaths were reasonably well‐predicted for seven of the nine seasons, we observed discrepancies for several age‐groups for 2011/2012 and 2012/2013; we note that in 2011/2012, the total estimated symptomatic incident cases were very low (Figure S4) compared with the total influenza‐attributable deaths (Figure 1), which underlies the lower model‐predicted values. Such discrepancies are due to factors associated with variation in incidence and/or influenza‐attributable mortality not present in the model. To reconstruct respiratory deaths, we assumed that the seasonal pattern in the proportion of total deaths with a respiratory cause was stable across seasons; this may have led to slight under‐ or over‐estimation of the modelled weekly numbers of influenza‐attributable deaths. Finally, and importantly, the 95% CrIs for cfrs reflect only uncertainty in the model and are consequently too narrow; as described in the Section 2, inherent uncertainty in symptomatic incidence and influenza‐attributable mortality could not be incorporated.

Our results depend crucially on unbiased and accurate measurement of the number of symptomatic infections, the distribution of circulating subtypes/lineages and the number of influenza‐attributed deaths per season. For instance, if mortality had been underestimated, or symptomatic incidence over‐estimated, then actual cfrs would be higher than we have inferred. Due to the rise in COVID‐19 mortality and COVID‐19‐related disruption in health‐care seeking behaviour, we truncated the estimation of both influenza‐attributable mortality and symptomatic influenza incidence for season 2019/2020 at Week 11. This truncation may have led to underestimation of both influenza incidence and influenza mortality in this season. However, the implementation of drastic public health measures early on—in Week 12 of 2020—to reduce contact rates^39^ are suspected to have led to much lower attack rates for other respiratory infections, including influenza (no influenza positives were detected after Week 11 in virological testing of swabbed sentinel GP network patients^40^), which suggests that underestimation was minimal.

The data sources underlying our symptomatic incidence estimates are from the general, non‐institutionalised population, but mortality data are from the entire population, which could lead to over‐estimation of the cfrs for institutional residents with a higher underlying mortality rate. However, given that only about 10% of persons aged 65+ years live in nursing or elderly homes,^41^ the impact on influenza incidence and consequently on case‐fatality estimates is expected to be small for all but the oldest age‐groups.

In summary, we estimated age‐specific cfrs for A(H3N2), A(H1N1)pdm09 and influenza B in a Bayesian inferential modelling framework. These cfrs can be used to estimate influenza‐attributable mortality in comparable settings for which data on (or estimates of) the incidence of cases and the season‐dependent proportions of circulating influenza (sub)types are available. Estimates thus reflect the situation in a country with accessible healthcare and free‐of‐charge influenza vaccination for risk groups; in the Netherlands, person aged 60 years and older and persons at increased risk of complications or mortality (e.g., chronic heart disorders, diabetes, immunodeficiency and nursing home residents) are eligible^42^; over the period 2011–2019, vaccination uptake varied between 50% and 66%, with higher uptake consistently observed for the age‐group 65+ years compared with 60–64 years.^42^ If the epidemiological situation changes, for instance due to the introduction of new vaccines with improved effectiveness, or to changes in vaccination coverage, cfrs can be easily re‐estimated. Estimates of the influenza‐attributable cfr per age‐group and sub(type) are vital for public health decision‐making—in which the allocation of finite resources may be based on annual health burden—for assessing the retrospective and prospective value of preventative interventions, for the modelling of control strategies including vaccination and for health economic evaluations.

## AUTHOR CONTRIBUTIONS

**Scott A McDonald:** Conceptualization; formal analysis; methodology; writing—original draft; writing—review and editing. **Anne C Teirlinck:** Conceptualization; formal analysis; writing—original draft; writing—review and editing. **Mariette Hooiveld:** Investigation; resources; writing—review and editing. **Liselotte van Asten:** Formal analysis; investigation; writing—review and editing. **Adam Meijer:** Investigation; resources; writing—review and editing. **Marit de Lange:** Data curation; investigation; writing—review and editing. **Arianne B van Gageldonk‐Lafeber:** Investigation; project administration; writing—review and editing. **Jacco Wallinga:** Formal analysis; methodology; project administration; writing—review and editing.

## CONFLICT OF INTEREST STATEMENT

The authors declare that they have no competing interests.

### ETHICS APPROVAL STATEMENT

This statistical modelling study did not require ethical approval, as stated by the Dutch medical ethics committee (document reference: WAG/om/15/030372).

### PEER REVIEW

The peer review history for this article is available at https://www.webofscience.com/api/gateway/wos/peer‐review/10.1111/irv.13146.

## Supporting information


**Figure S1.** Log‐transformed case fatality ratios per 5‐year age‐group (filled circles), based on additive regression‐modelled influenza‐attributable mortality and estimated symptomatic incidence. Data are aggregated over seasons 2011/2012 through 2019/2020. Dashed lines indicate piecewise log‐linear fits. Line segments indicate 95% confidence intervals around estimated log case‐fatality ratios.
**Figure S2**. Additive regression‐modelled influenza‐attributable deaths compared with predicted influenza deaths (calculated using inferred case‐fatality ratios), per season and age‐group (55–59 through 85 + years). Line segments indicate 95% credible intervals.
**Figure S3**. Observed age‐distribution of influenza deaths based on ICD‐10 codes among persons aged <55 years, as used in the Burden of Communicable Diseases in Europe (BCoDE) project (Cassini et al., 2018). Distribution was derived by averaging over data from four countries, over the four‐year period 2005–2008.
**Figure S4**. Estimated incidence symptomatic influenza per season and broad age‐group, for seasons 2011/2012 through 2019/2020. Table below the plot indicates the (sub)type‐specific proportion of a positive result (aggregating over age) from laboratory testing of sampled ILI patients from sentinel GP surveillance (see Methods).
**Figure S5**. Directed acyclic graph of the statistical model for influenza (sub)type specific case‐fatality ratios for age‐groups 55–59 years through 85 + years. Rectangles indicate data/constants and circles indicate parameters, with double circles for those parameters for which priors are specified. Solid and dashed arrows indicate stochastic and functional relationships, respectively.
**Code S1**. JAGS code for inference of influenza (sub)type‐specific case‐fatality ratios.
**Code S2**. JAGS code for estimating age‐dependent case‐fatality ratios and accompanying 95% confidence intervals, aggregating over influenza (sub)type.

## Data Availability

The data required to estimate the principal outputs of this study that are not already provided in tables and/or figures can be obtained upon reasonable request from the corresponding author**.**
