# Supplementary material for: Inference of age‐dependent case‐fatality ratios for seasonal influenza virus subtypes A(H3N2) and A(H1N1)pdm09 and B lineages using data from the Netherlands
Source: Influenza Other Respir Viruses. 2023 Jun 19;17(6):e13146. doi: 10.1111/irv.13146 (PMC10279999; doi:10.1111/irv.13146)
Supplement: Supplementary file 1 — Figure S1. Log‐transformed case fatality ratios per 5‐year age‐group (filled circles), based on additive regression‐modelled influenza‐attributable mortality and estimated symptomatic incidence. Data are aggregated over seasons 2011/2012 through 2019/2020. Dashed lines indicate piecewise log‐linear fits. Line segments indicate 95% confidence intervals around estimated log case‐fatality ratios. Figure S2. Additive regression‐modelled influenza‐attributable deaths compared with predicted influenza deaths (calculated using inferred case‐fatality ratios), per season and age‐group (55–59 through 85 + years). Line segments indicate 95% credible intervals. Figure S3. Observed age‐distribution of influenza deaths based on ICD‐10 codes among persons aged <55 years, as used in the Burden of Communicable Diseases in Europe (BCoDE) project (Cassini et al., 2018). Distribution was derived by averaging over data from four countries, over the four‐year period 2005–2008. Figure S4. Estimated incidence symptomatic influenza per season and broad age‐group, for seasons 2011/2012 through 2019/2020. Table below the plot indicates the (sub)type‐specific proportion of a positive result (aggregating over age) from laboratory testing of sampled ILI patients from sentinel GP surveillance (see Methods). Figure S5. Directed acyclic graph of the statistical model for influenza (sub)type specific case‐fatality ratios for age‐groups 55–59 years through 85 + years. Rectangles indicate data/constants and circles indicate parameters, with double circles for those parameters for which priors are specified. Solid and dashed arrows indicate stochastic and functional relationships, respectively. Code S1. JAGS code for inference of influenza (sub)type‐specific case‐fatality ratios. Code S2. JAGS code for estimating age‐dependent case‐fatality ratios and accompanying 95% confidence intervals, aggregating over influenza (sub)type. [file IRV-17-e13146-s001.docx]

**SUPPLEMENTARY MATERIALS**

*To accompany the paper entitled:*

Inference of age-dependent case-fatality ratios for seasonal influenza virus subtypes A(H3N2) and A(H1N1)pdm09 and B lineages using data from the Netherlands

*Authors:*

Scott A McDonald^1^, Anne C Teirlinck^1^, Mariette Hooiveld^2^, Liselotte van Asten^1^, Adam Meijer^1^, Marit de Lange^1^, Arianne B van Gageldonk-Lafeber^1^, Jacco Wallinga^1,3^

^1^ Centre for Infectious Disease Control, National Institute for Public Health and the Environment (RIVM), PO Box 1, 3720 BA Bilthoven, Netherlands

^2^ Nivel, Utrecht, Netherlands

^3^ Department of Biomedical Data Sciences, Leiden University Medical Center, Leiden, Netherlands


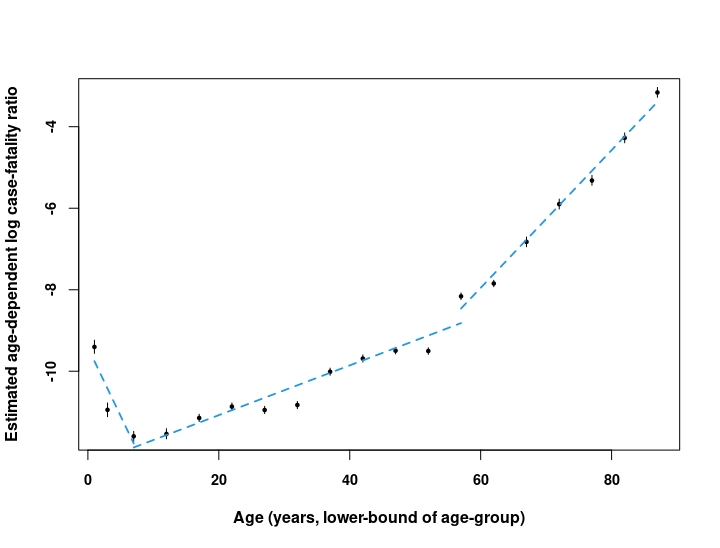
**Fig. S1**. Log-transformed case fatality ratios per 5-year age-group (filled circles), based on additive regression-modelled influenza-attributable mortality and estimated symptomatic incidence. Data are aggregated over seasons 2011/2012 through 2019/2020. Dashed lines indicate piecewise log-linear fits. Line segments indicate 95% confidence intervals around estimated log case-fatality ratios.

**Fig. S2**. Additive regression-modelled influenza-attributable deaths compared with predicted influenza deaths (calculated using inferred case-fatality ratios), per season and age-group (55-59 through 85+ years). Line segments indicate 95% credible intervals.


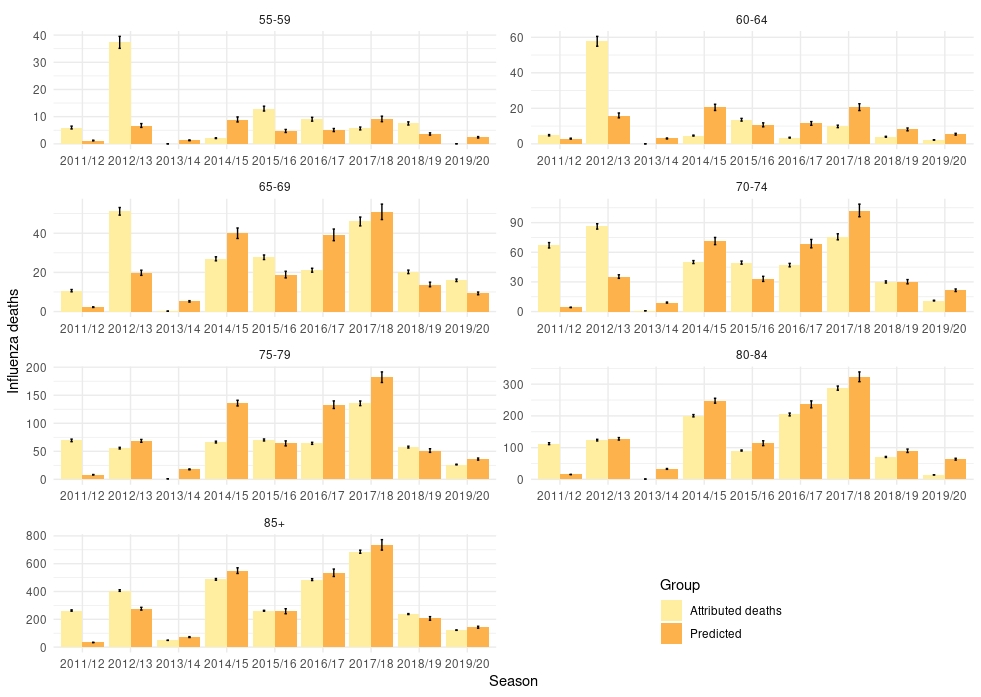


**Fig. S3**. Observed age-distribution of influenza deaths based on ICD-10 codes among persons aged <55 years, as used in the Burden of Communicable Diseases in Europe (BCoDE) project (Cassini et al., 2018). Distribution was derived by averaging over data from four countries, over the four-year period 2005-2008.


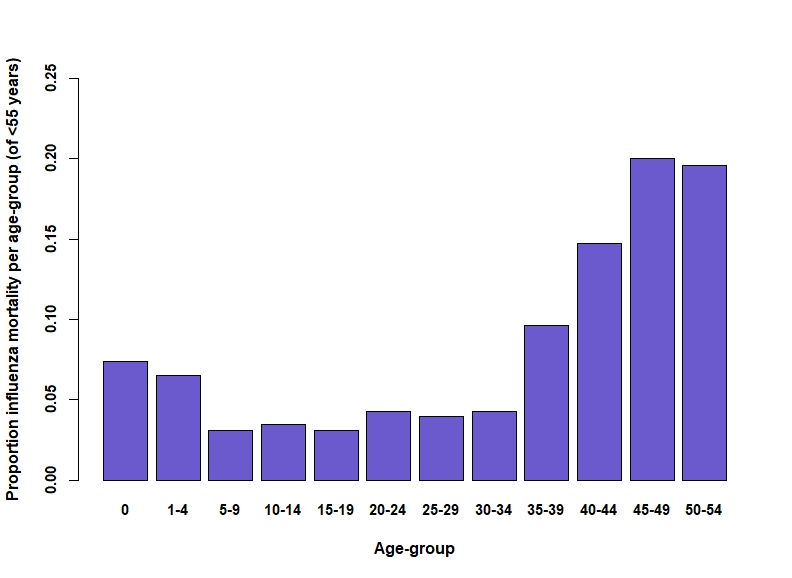


**Fig. S4**. Estimated incidence symptomatic influenza per season and broad age-group, for seasons 2011/2012 through 2019/2020. Table below the plot indicates the (sub)type-specific proportion of a positive result (aggregating over age) from laboratory testing of sampled ILI patients from sentinel GP surveillance (see Methods).


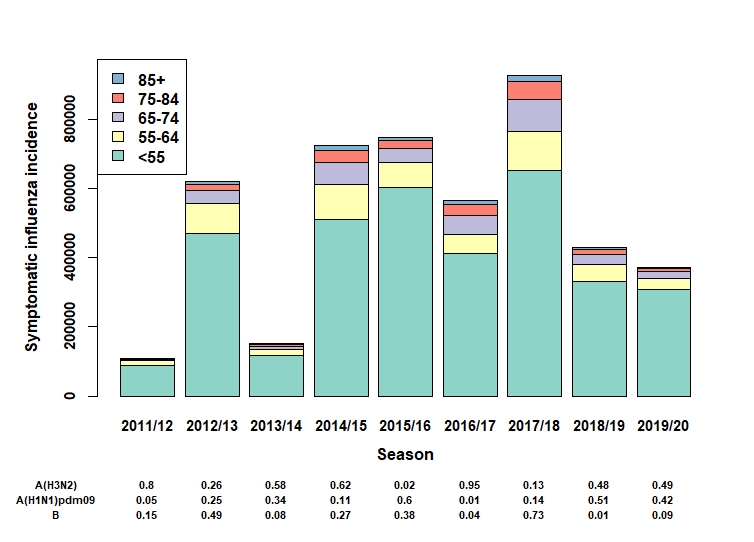


**Fig. S5**. Directed acyclic graph of the statistical model for influenza (sub)type specific case-fatality ratios for age-groups 55-59 years through 85+ years. Rectangles indicate data/constants and circles indicate parameters, with double circles for those parameters for which priors are specified. Solid and dashed arrows indicate stochastic and functional relationships, respectively.


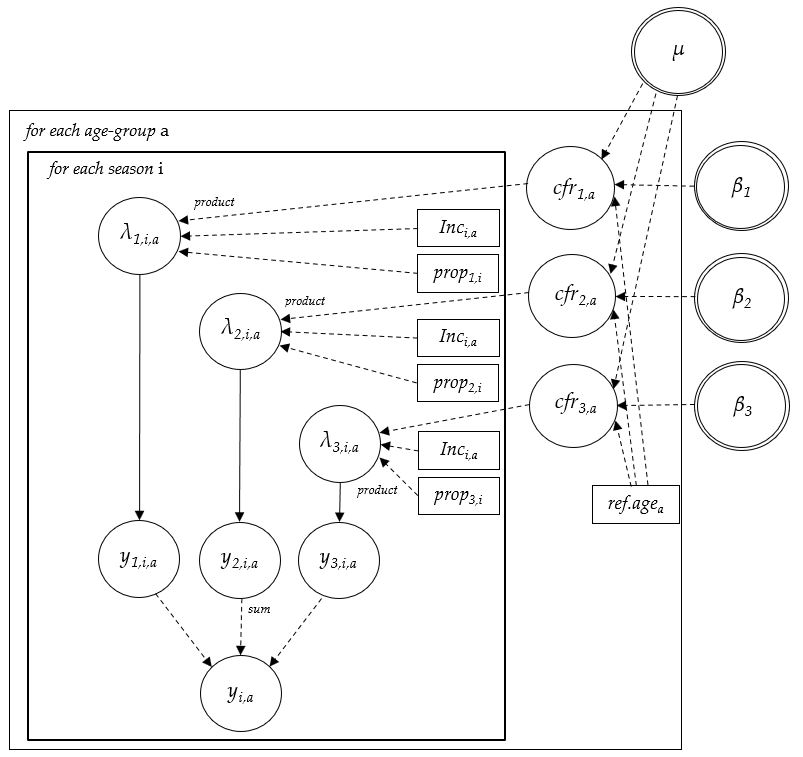


**Code S1**. JAGS code for inference of influenza (sub)type-specific case-fatality ratios.

model {

# Priors

Mu ~ dnorm(0,0.001) # common age-effect on case-fatality ratios

Beta1 ~ dnorm(0,0.001) # intercept, subtype H3N2

Beta2 ~ dnorm(0,0.001) # intercept, subtype H1N1

Beta3 ~ dnorm(0,0.001) # intercept, influenza B

# Likelihood

for(a in 1:N) {

for(i in 1:NS) {

y1[i,a] ~ dpois(lambda1[i,a]) # Eq. 2

lambda1[i,a] <- cf1[a]*prop1[i]*inc[i,a] # Eq. 3

y2[i,a] ~ dpois(lambda2[i,a])

lambda2[i,a] <- cf2[a]*prop2[i]*inc[i,a]

y3[i,a] ~ dpois(lambda3[i,a])

lambda3[i,a] <- cf3[a]*prop3[i]*inc[i,a]

y[i,a] ~ sum(y1[i,a],y2[i,a],y3[i,a]) # Eq. 1

# Predicted deaths per season & age-group, aggregating over (sub)type

y.pred[i,a] <- (cf1[a]*prop1[i]*inc[i,a]) + (cf2[a]*prop2[i]*inc[i,a]) +

(cf3[a]*prop3[i]*inc[i,a])

}

log(cf1[a]) <- Betal + Mu * ref.ag[a] # Eq. 4

log(cf2[a]) <- Beta2 + Mu * ref.ag[a]

log(cf3[a]) <- Beta3 + Mu * ref.ag[a]

}

# Nodes for total influenza deaths per (sub)type, aggregated over season and age-group

y1.all <- sum(y1[,])

y2.all <- sum(y2[,])

y3.all <- sum(y3[,])

y.all <- (sum(y1[,]) + sum(y2[,]) + sum(y3[,]))

cf2.65plus <- sum(y2[,3:N])/sum(inc2[,3:N]) # ad-hoc node for H1N1 cfr, 65+ yrs only

}

data <- list(

NS = 9, # no. seasons = 9

N = 7, # no. agegrps in analysis (55-59 ... 85+)

ref.ag = c(0,5,10,15,20,25,30), # 0 corresponds to 55-59 years... 30 to 85+ years

inc = SymInc, # pre-calculated symptomatic incidence, 9 x 7 matrix

prop1 = prop_H3, # per-season proportion H3N2-positive among all tested samples

prop2 = prop_H1, # per-season proportion H1N1-positive among all tested samples

prop3 = prop_B, # per-season proportion infl-B positive among all tested samples

inc2 = SymInc*prop_H1, # needed only for ad-hoc 65+ yrs estimate

y = round(InflDths) # pre-calculated influenza-attribuable deaths, 9 x 7 matrix

)

**Code S2**. JAGS code for estimating age-dependent case-fatality ratios and accompanying 95% confidence intervals, aggregating over influenza (sub)type.

model {

for(a in 1:N) {

for(i in 1:NS) {

inc.prop[i,a] ~ dbeta(inc.beta.a[i,a],inc.beta.b[i,a])

inc[i,a] <- inc.prop[i,a]*inc.pop[i,a]

dths[i,a] ~ dnorm(dths.m[i,a],1/dths.sd[i,a]^2)

}

inc.a[a] <- sum(inc[1:NS,a])

dths.a[a] <- sum(dths[1:NS,a])

cf.a[a] <- sum(dths[1:NS,a])/sum(inc[1:NS,a]) # age-group dependent case-fatality ratio

}

inc.all <- sum(inc.a[1:N]) # all-ages

dths.all <- sum(dths.a[1:N]) # all-ages

cf.all <- sum(dths.a[1:N])/sum(inc.a[1:N]) # all-ages case-fatality ratio

younger55 <- sum(dths.a[1:12]) # deaths in <55 years only

}

data <- list(

NS = 9, # no. seasons = 9

N = 19, # no. age-groups = 19

dths.m = InflDths_m, # mean no. infl-attributable deaths, 9 x 19 matrix

dths.sd = InflDths_sd, # SD of infl-attributable deaths, 9 x 19 matrix

inc.beta.a = SymInc_a, # Beta a & b params for inc proportion, 9 x 19 matrix

inc.beta.b = SymInc_b,

inc.pop = SymInc_pop # popul size for inc proportion, 9 x 19 matrix

)
